# Reviews

**Published:** 1880-09

**Authors:** 


### Reviews.

---

**Common Mind-Troubles and the Secret of a Clear Head.** By J. MORTIMER GRANVILLE, M. D. Philadelphia: D. G. Brinton, Publisher.

It is unfortunate that the author did not make his professional *debut* here, in a more comprehensive work on the same subject. Many of the underlying ideas are excellent, but in the effort to present them in a small space and in popular style, they are apt to appear dwarfed and distorted.

Under the head of common mind-troubles, he treats of "failings" and "defects of memory," considers "confusions of thought" and gives good hints for avoiding "sleeplessness from thought," seasoning these and similar chapters with generous sprinkling of wholesome advice. "The secret of a clear head" may still remain an unanswered conundrum to those who have finished this part of the book, but the writer at least delivers from this text some short but excellent homilies which will be appreciated by many readers. L. H.

---

**Lucie Rodey.** A Novel by HENRY GREVILLE Translated by Mary Neal Sherwood Philadelphia: T. B Peterson & Bro.

The publishers are very considerate to offer a book like this to prosaic doctors. Such special favor deserves more than a simple "acknowledgment," for although if we are not addicted to literature of this kind, it has its uses none the less. When medical students grow drowsy over Gray and Flint, a racy novel would stir them up afresh, and even in some cases of suspended animation, where no effect can be obtained from artificial respiration, nitrite of amyl or from electricity, it may be well to try the reading of a chapter from Lucie Rodey. L. H.

**A Hand-Book of Physical Diagnosis**, comprising the Throat, Thorax and Abdomen By DR. PAUL GUTTMAN, Privat Docent in Medicine, University of Berlin. Translated from the Third German Edition by Alex. Napier, M.D., Fel. Fac. Phys. and Surg., Glasgow. With a colored plate and eighty-nine fine wood engravings. New York: William Wood & Co. 1880.

This valuable hand-book on Physical Diagnosis has been received with such favor by the profession on the Continent as to be translated into several modern languages, and also to be chosen for the new Sydenham Society Publications for 1879. These facts demonstrate the reputation of the work, and its importance in the special department of which it treats.

It aims to present a concise description of the different methods adopted in the clinical examination of the thoracic and abdominal viscera in health and disease, to which is added a chapter on the examination of the Larynx. The enterprising publishers have shown excellent judgment in adding this work to the "Library Series." It is full and complete, yet concise in its descriptions, and contains in a condensed form, suited to the general practitioner, the methods necessary to be adopted in the physical diagnosis of disease. L.

---

**Lessons in Gynecology.** By WILLIAM GOODELL, A. M., M. D., Professor of Clinical Gynecology, in the University of Pennsylvania, &c. With ninety-two Illustrations. Philadelphia: D. G. Brinton, 115 South Seventh Street. 1880.

Dr. Goodell, in the preface to the first edition, states that this book is not a treatise upon the diseases of women, but mainly the outcome of clinical and didactic lectures. Herein the author furnishes the clue to the real value of the important work he has given the profession. It is a practical treatise upon many of the diseases and accidents peculiar to women, and both the matter and the style in which it is presented, is eminently characteristic of the author. Dr. Goodell uses no superfluous words to express his meaning, and drives directly at the point at issue in a truly American manner. While therefore the work he presents does not show the erudition of other authors, it is so intensely practical that its value to the general practitioner is greatly enhanced, and its importance for reference correspondingly increased. To the specialist the work is very useful, inasmuch as it gives the experience and views of one of the most practical of American gynecologists, while it furnishes data upon various subjects daily met with in practice. We regard the work as an important and valuable contribution to gynecological literature.

L.

---

**The Surgery; Surgical Pathology and Surgical Anatomy of the Female Pelvic Organs.** In a series of plates taken from nature, with commentaries, notes and cases. By HENRY SAVAGE, M. D., London, Fellow of the Royal College of Surgeons of England, etc. Third edition, revised and greatly extended. 32 plates and 22 wood engravings, with special illustrations of the operations on Vesico-Vaginal, Fistula, Ovariotomy, and Perineal operations. New York: William Wood & Co. 1880.

In including this valuable English work on the surgery, surgical pathology of the female pelvic organs in their library series, Messrs. Wood & Co. have shown commendable enterprise, and placed the profession under lasting obligations by offering, at a very moderate cost, a guide to the study of a class of diseases and accidents, which are only too frequently met with of late. The illustrations are numerous and wonderfully accurate, and give in a small compass not only the anatomy, but the surgical relations of this important portion of the human organism. The text is devoted to the explanation of the plates with references to the histological structure and composition of the parts. It also takes up the pathology and pathological anatomy of the uterus and contiguous organs, with admirable plates illustrating vesico-vaginal fistula, uterine displacements, vagino-cystocele, rectocele, with two plates showing the successive steps in the operation for ovariotomy. The work is eminently practical, abundantly illustrated, and from the well-known reputation of the author claims the hearty endorsement of the profession. Its value is alone more than the entire cost of the series.

